# The associations between ADHD and asthma in Korean children

**DOI:** 10.1186/1471-244X-14-70

**Published:** 2014-03-10

**Authors:** Ho Jang Kwon, Mi Young Lee, Mina Ha, Seung Jin Yoo, Ki Chung Paik, Jong-Han Lim, June Sakong, Chul-Gab Lee, Dong-Muk Kang, Soo Jong Hong, Hwan Il Cho, Myung Ho Lim

**Affiliations:** 1 Environmental Health Center, Dankook University Hospital, Cheonan, Korea; 2 Department of Preventive Medicine, College of Medicine, Dankook University, Cheonan, Korea; 3 Department of Preventive Medicine, College of Medicine, Keimyung University, Daegu, Korea; 4 Department of Psychology and Psychiatry, College of Medicine, Dankook University, 359 Manghyang Rho, Cheonan 300-714, South Korea; 5 Department of Preventive Medicine, College of Medicine, Inha University, Incheon, Korea; 6 Department of Preventive Medicine, College of Medicine, Yeongnam University, Daegu, Korea; 7 Department of Preventive Medicine, College of Medicine, Chosun University, Kwangju, Korea; 8 Department of Preventive Medicine, College of Medicine, Pusan University, Pusan, Korea; 9 Department of Pediatrics, College of Medicine, Ulsan University, Ulsan, Korea

**Keywords:** ADHD, Asthma, Allergic rhinitis, Children

## Abstract

**Background:**

The aim of this study was to investigate the associations between attention deficit hyperactivity disorder (ADHD), the most common neuropsychiatric disorder in school children, and childhood allergic disease by evaluating their respective prevalence.

**Methods:**

Subjects were comprised of first and second grade students in twenty two elementary schools in a city in the Republic of Korea. The mode of measurement for ADHD was based on DSM-IV from clinical interviews conducted by child psychiatrists. Along with the diagnostic interviews, we also used the epidemiological questionnaires, Computerized Attention Deficit-Hyperactivity Disorder Diagnostic System, the abbreviated Conner’s Parent Rating Scale (CPRS), and DuPaul’s ADHD Rating Scales. Allergic conditions, such as asthma, have been separately evaluated based on the questionnaire items whose validity and reliability were proved by the International Study of Asthma and Allergies in Children (ISAAC). All questionnaires were completed by the subjects’ parents.

**Results:**

The lifetime prevalence rate of asthma in ADHD patients was 36.6%, compared to a prevalence of 24.3% in control subjects. The lifetime prevalence rate of allergic rhinitis in ADHD patients was 59.0%, compared to a prevalence of 47.0% in control subjects. Statistically significant difference has been found between the two groups. In the logistic regression model of the ADHD and the control group, the relative risk of asthma was 1.60 times higher (confidence interval 1.301-1.964), the relative risk of allergic rhinitis was 1.38 times higher (confidence interval 1.124-1.681), which showed statistical significance.

**Conclusions:**

The findings of this study suggest significant association between ADHD and childhood asthma and allergic rhinitis. Therefore, appropriate evaluation and treatment are needed for asthmatic children with attention-deficit symptoms, or allergic rhinitis with ADHD. Besides, further research is needed to determine the etiological approach towards ADHD, asthma, and allergic rhinitis.

## Background

Asthma and other allergic disease are common childhood chronic medical illness estimated to affect 7 to 40% of children [1]. The characteristic symptoms of asthma include coughing, wheezing, shortness of breath, and chest discomfort, pain, or pressure. Also the characteristic symptoms of allergic rhinitis are rhinorrhea (excess nasal secretion), itching, and nasal congestion and obstruction. The prevalence rate of asthma and other allergic disease for children aged from 5 to 14 years had dramatically increased up to 74% between 1980 and 1994 [2]. Recently, it has been suggested that asthma and other allergic disease may be a risk factor for attention-deficit hyperactivity disorder that cause physical and emotional problems, long-term school absence, and social problems.

Attention Deficit Hyperactivity Disorder (ADHD) is a common child psychiatric disorder characterized by cognitive problems, such as attention deficit, and behavioral problem, such as hyperactivity and impulsivity [3]. ADHD is a very common neuropsychiatric disorder the prevalence of which is 2-7.6% among children of school age in South Korea [4,5]. Genetic factor is considered to be the strongest among the causes of ADHD. However, it is recently considered that the cause may be more complex as not only genetic factor is involved but environmental factors are also implicated [6,7].

Some of the recent studies suggest that school and behavioral problems, or ADHD symptoms are caused by asthma and its treatment [8,9], while other studies show no associations between these factors [10,11]. Despite contradictory results, general observations appear to suggest an association between some allergic disease and ADHD.

Asthmatic and other allergic children usually experience difficulties with academic performance compared to their non-asthmatics. This chronic illness or its treatment may cause various conflicts in their school life, due to their physical and emotional problems they face, which results in negative impacts to their quality of life. According to Kashani et al. [12], 63% of 56 asthmatic children met the diagnostic criteria for psychiatric disorders. Bussing et al. [13] also found that children with severe asthma were 3 times more likely to demonstrate severe behavioral problems compared to children without chronic conditions.

Asthmatic and allergic children may experience attention problems exacerbated by hypoxia and actions to maintain their breaths. Besides, breathlessness at night can create sleep disturbance, further exacerbating attention deficit hyperactivity disorder and impulsive behaviors in asthmatic and allergic children would be the secondary effects of allergic disease and its treatment. According to parents’ reports in some studies, asthmatic and allergic children were more likely to have academic and behavior problems than those without allergic disease [8,9]. A Korean study demonstrated that children with allergic conditions are more likely to have difficulty socializing, and experience emotional and behavioral problems compared to those without [14]. Among many emotional and behavioral problems, attention deficit particularly affects children’s psychosocial adjustment, including their ability to socialize, interpersonal relationships, unrest, and mood control [15].

Contrary to a study finding that neurobehavioral disorder is secondary to allergic disease and its treatment, other studies reported no associations between neurobehavioral disorder regarding school and behavioral problems and allergic disease or its treatment [10,11].

According to the recent study of Blackman et al. [16], which surveyed the parents of 102,353 children aged between 0 and 17 years, more asthmatic children suffered ADHD, depression, conduct disorders, learning disabilities, and absenteeism. The more severe the symptoms of asthma, the more common were these problems.

Asthma and allergic disease medication is known to cause side effects including creating restlessness, sleep disturbances, headache, or emotional and mood changes [17,18]. Pretorius et al. [19] suggested that serotonin played a major role in the pathophysiology of asthma, and asthma medication lowered serotonin levels in the brain, which results in symptoms symptoms similar to ADHD.

The aim of this study was to determine the association between allergic disease and ADHD by evaluating the prevalence of allergic disease in children in the first and second grade in three elementary schools. In addition, this study further examined the association between allergic disorders and ADHD.

## Methods

### Study design, participants and procedure

A complete enumeration was performed in this study, we collected the data all year long. Total response rate was 75 percentile. The subjects were first and second grade children in twenty two elementary schools located in seven cities (Yeosu, Incheon, Pusan, Mokpo, Cheonan, Cheju, Cheongeup). Yeosu, Incheon, and Pusan were industrial cities. Mokpo, Cheonan, Cheju, and Cheongeup were rural area Questionnaires were distributed to the children’s parents, who completed them before being collected them at the schools. The total number of control subjects was 3,564 children, of whom 1704 were boys (47.9%) and were 1,857 girls (52.1%), the total number of ADHD subjects was 549 children, of whom 413 were boys (75.2%) and were 136 girls (24.8%) (Table 1).

**Table 1:** Demographic data of ADHD children group (N = 549) and control group (N = 3,564)

**Variables**	**Control group (%)**	**ADHD group (%)**	**F**	**p value**	
**Age**^**a**^	7.78 ± 1.17	7.83 ± 1.20	4.476	.342	
**Gender**^**b**^			1,131.268	.000	
Male	1,704(47.9%)	413(75.2%)			
Female	1,857(52.1%)	136(24.8%)			

These data represent mean ± S.D., by independent t test^a^, or N(%), by chi-square test^b^, significant p value <0.05, ADHD: attention deficit hyperactivity disorder.

ADHD was diagnosed according to the DSM-IV criteria, based on interviews with a child psychiatrist. Along with the diagnostic interviews, we also used epidemiological questionnaires, Tests of Variable Attention computer program (TOVA), the abbreviated Conner’s Parent-Teacher Rating Scale (CPRS), and DuPaul’s ADHD Rating Scales. The purpose of the study was explained to all participants, including parents, and caretakers, and the study was conducted with consent obtained from the participants. Informed consents were obtained from children and their parents prior to study entry. The study was also approved by Dankook Hospital Ethics Committee.

### Measurements and assessment

#### Epidemological questionnaires

The items of the questionnaire include participants’ gender, age, grade, past records of disease, and developmental characteristics. To all subjects a complete enumeration was conducted. The evaluation for asthma and the allergic disorders was based on the items defined by the International Study of Asthma and Allergies in Children (ISAAC) [1], of which the reliability and validity were proven. All questionnaires were completed by the parents or caretakers.

#### Asthma

The prevalence was evaluated based on four questions: 1. Subjects were considered to have asthmatic symptoms if wheezing or whistling was experienced. The questionnaire also included questions to delineate their experience of wheezing or whistling in the chest for the past 12 months, if they have ever been diagnosed with asthma, and if they have ever received treatment due to asthma for the past 12 months.

#### Atopic dermatitis

If itchy skin rashes had been frequently experienced for at least 6 months, it would be considered a symptom of atopic dermatitis. The questionnaire items further delineated if the subjects had ever experienced itchy skin rashes for the past 12 months, if they had ever been diagnosed with eczema (“congenital fever” or “atopic dermatitis”), and if they had received treatment for eczema (“congenital fever” or “atopic dermatitis”) for the past 12 months. The prevalence of atopic dermatitis was determined based on the above questions.

#### Allergic rhinitis

Allergic rhinitis was considered to be present if the subject had experienced repetitive sneezing, rhinorrhea, or nasal congestion when they did not get cold or flu. The questionnaire items further delineated if they had ever experienced repetitive sneezing, rhinorrhea, or nasal congestion for the past 12 months, if they had ever been diagnosed with allergic rhinitis, and if they had ever been treated for allergic rhinitis for the past 12 months. The prevalence of allergic rhinitis was determined based on the above questions.

#### Allergic conjunctivitis

Allergic conjunctivitis was considered to be present if participants experienced frequent itchy eyes when they did not have Apollo Conjunctivitis (epidemic conjunctivitis). The questionnaire items further delineated if they had experienced frequent itchy eyes for the past 12 months, if they had been diagnosed with allergic conjunctivitis, and if they had ever been treated for allergic conjunctivitis for the past 12 months. The prevalence of allergic conjunctivitis was evaluated based on the above questions.

#### Food allergy

Food allergy was considered to be present if participants had shown allergic symptoms towards certain food. The questionnaire items further delineated if they had shown allergic symptoms towards certain food, if they had ever been diagnosed with food allergy, and if they had ever been treated for food allergy for the past 12 months. The prevalence of food allergy was determined based on the above questions.

#### Computerized attention deficit-hyperactivity disorder diagnostic system (ADS)

ADS, a modified test of Variable Attention computer program (TOVA) in Korea, is a computerized program being widely used to diagnose ADHD and assess continuous performance function in children with attention-deficit or hyperactivity. As one of the most popular Continuous Performance Tests (CPT) in Korea, ADS was developed and standardized by Shin et al. [20]. It is divided into visual and auditory tests. ADS is used to assess general attention ability and its detailed evaluation index includes omission errors, commission errors, hit reaction time mean, hit reaction time standard deviation, attentiveness, and risk taking. Omission errors indicate the number of times the target is presented but the subject fails to respond. It measures inattentiveness. Commission errors indicate the number of times responses are given to non-targets and it measures the extent of response prevention and impulsivity. Hit reaction time mean is the average speed of correct response and it measures the speed of response or information processing. Hit reaction time deviation is the standard deviation of hit reaction time and it measures response speed consistency and attention flexibility. A score of above 70 in omission errors, commission errors, or hit reaction time standard deviation is a represent of continuous attention deficit and that clinically attention deficit hyperactivity disorder (ADHD) is suspected.

#### Abbreviated conners parent-teacher rating scale (revised)

Goyette et al. replicated and designed a 10-item index from the Conner’s Parent-Teacher Rating Scale, which was initially developed by Conners in 1970 to measure major symptoms of childhood ADHD [21]. In Korea, Oh reported its reliability in a study of domestic children [21]. The rating scale consists of 4 point values, completed by parents or teachers, indicating 0 for No symptoms, 1 for mild, 2 for moderate, and 3 for severe. All points are summated to obtain the total score. The range of score is 0 to 30 and ADHD children score above 16 points by parents or 17 points by teachers.

#### DuPaul’s ADHD rating scales

Being developed by DuPaul based on the DSM-IV diagnostic criteria for ADHD, DuPaul ADHD Rating Scales is used for both parent and teacher assessment for child behavior [22]. This Scales with 18 items is proven for its validity in differentiating children with ADHD from those without, and effective in differentiating three subtypes of ADHD: predominantly inattentive, predominantly hyperactive-impulsive, and combined hyperactive-impulsive and inattentive. Each item scores 1 to 3 and a score above 2 is considered abnormal in child development. Odd-numbered items measure inattentiveness and even-numbered items measure hyperactivity and impulsivity. Domestic standardization was performed by Kim et al. [22] and children with ADHD may score above 19 by parents or/and 18 by teachers.

### Statistical analysis

SPSS 15.0 was used to process data, and cross-tabulations analyses were performed to analyze epidemiological questionnaires when needed. Independent T-test was performed to compare scores of allergic history between the ADHD and control subjects. To examine the association between ADHD and allergic disorders, logistic regression analysis was performed on ADHD data and allergic lifetime history. In each case, it is considered to be statistically significant when the *p*-value <0.05.

## Results

### Social demographic characteristics of the participants

Participants were divided into the ADHD group (*n =* 549) and the control group (*n =* 3,564). Depending on whether participants suffer from allergy, questions on allergic history were classified into: asthma history, atopy history, allergic rhinitis history, allergic conjunctivitis, food allergy, and drug allergic history. The mean age of the ADHD group was 7.83 ± 1.20 years, comprised of 413 boys (75.2%) and 136 girls (24.8%). The mean age of the control group was 7.78 ± 1.17 years, comprised of 1,704 boys (47.9%) and 1,857 girls (52.1%). A difference in sex ratio of social-demographic characteristics between the two groups was statistically significant (Table 1).

### Prevalence rates of ADHD group and control group

The prevalence of allergic disorders in the ADHD group are as follows: 36.6% had asthma, 59.0% had allergic rhinitis, 41.4% had atopy, 29.3% had allergic conjunctivitis, 12.9% had food allergy, and 2.8% had drug allergy. The prevalence of allergic disorders in the control group are as follows: 24.3% had asthma, 47.2% had allergic rhinitis, 38.4% had atopy, 25.6% had allergic conjunctivitis, 11.6% had food allergy, and 2.0% had drug allergy (Table 2).

**Table 2:** Allergic history of ADHD children group (N = 549) and control group (N = 3564)

**Variables**		**Control group (%)**	**ADHD group (%)**	**F**	**p value**
**Asthma**	Lifetime	859(24.3)	200(36.6)	99.09	0.000
	12 months	428(12.1)	112(20.5)	100.17	0.000
	Diagnostic Hx	373(10.6)	86(15.8)	46.75	0.002
	Treatment Hx	153(4.3)	37(6.8)	24.70	0.031
**Allergic rhinitis**	Lifetime	1,643(47.2)	316(59.0)	56.08	0.000
	12 months	1,514(43.5)	301(56.2)	.01	0.000
	Diagnosis Hx	883(25.4)	162(30.2)	19.37	0.023
	Treatment Hx	755(21.7)	146(27.6)	28.06	0.007
**Atopy**	Lifetime	1,335(38.4)	221(41.4)	5.60	0.193
	12 months	867(24.9)	144(27.0)	3.78	0.316
	Diagnosis Hx	1,188(34.2)	184(34.5)	.06	0.899
	Treatment Hx	648(18.7)	106(19.9)	1.64	0.516
**Allergic conjunctivitis**	Lifetime	894(25.6)	158(29.3)	12.06	0.075
	12 months	569(16.3)	97(18.0)	3.86	0.331
	Diagnostic Hx	616(17.6)	105(19.5)	4.28	0.305
	Treatment Hx	365(10.5)	59(10.9)	.47	0.730
**Food allergy**	Lifetime	406(11.6)	69(12.9)	2.80	0.397
	12 months	224(6.4)	48(9.0)	18.52	0.050
	Diagnosis Hx	205(5.9)	35(6.6)	1.457	0.544
	Treatment Hx	102(2.9)	24(4.5)	14.50	0.098
**Drug allergy**	Lifetime	69(2.0)	15(2.8)	6.08	0.274
	12 months	32(0.9)	10(1.9)	16.06	0.118
	Diagnosis Hx	30(0.9)	10(1.9)	18.95	0.097
	Treatment Hx	13(0.4)	7(1.3)	32.46	0.064

These data represent N(%) by chi-square test, significant p value <0.05, ADHD: attention deficit hyperactivity disorder, Hx:history.

Difference in the prevalence of asthma between the ADHD and the control group was statistically significant (F = 99.09, *p =* 0.000). Also difference in the prevalence of allergic rhinitis between the ADHD and the control group was statistically significant (F = 56.08, *p =* 0.000). The prevalence of other allergic disorders did not show statistically significant difference between the two groups (Table 2).

Twenty point five percent in the ADHD group, and 12.1% of the control group with asthma history received treatment during the past 12 months. The difference did not reach statistical significance, but the ADHD group were more likely to have received treatment during the past 12 months (F = 100.17, *p =* 0.000). Fifty six point two percent of the ADHD group and 43.5% of the control group with allergic rhinitis during the past 12 months, which was statistically significant (F = 0.02, *p =* 0.000). Other categories of allergic disorders did not show statistical significance between the two groups regarding the participants’ treatment history (Table 2).

### Relative risk of the ADHD group and the control group

In the logistic regression model of the ADHD and the control group, the relative risk of asthma life history was 1.60 times higher (confidence interval 1.301-1.964), which showed statistical significance (Chi-square = 19.90, *p =* 0.000), the relative risk of allergic rhinitis life history was 1.38 times higher (confidence interval 1.124-1.681), which showed statistical significance (Chi-square = 9.604, *p =* 0.002) (Table 3).

**Table 3:** Parameter estimates for logistic model of ADHD children group and control group

**Variables**	**Parameter estimate**	**Standard error**	**Chi-square**	**p value**	**Odds ratio**
**Gender**	-1.137	0.108	111.302	0.000	0.321(0.260-0.396)
**Age**	-0.072	0.040	3.310	0.069	0.930(0.860-1.006)
**Asthma life history**	0.469	0.105	19.915	0.000	1.598(1.301-1.964)
**Allergic rhinitis life history**	0.318	0.103	9.604	0.002	1.375(1.124-1.681)
**Atopy life history**	0.057	0.103	0.302	0.583	1.058(0.864-1.296)
**Allergic conjunctivitis life history**	-0.029	0.112	0.068	0.795	0.971(0.779-1.211)
**Food allergy life history**	-0.066	0.152	0.191	0.662	0.936(0.695-1.260)
**Drug allergy life history**	0.138	0.311	0.195	0.659	1.147(0.624-2.111)

These data represent by chi-square test, significant p value <0.05, ADHD: attention deficit hyperactivity disorder.

## Discussion

This study demonstrated the prevalence of asthma to be 36.6% in the ADHD group and 24.3% in the control group, and the difference between the two groups was statistically significant (F = 99.09, p = 0.000). The International Study of Asthma and Allergies in Childhood (ISAAC) questionnaires have shown that the prevalence of childhood asthma is increasing worldwide. The prevalence of ‘asthma ever’ was 17.0% in 1995 for elementary school students [23]. Our sample showed the similar result as the previous prevalence study in Korea. This result is aligned with the result of Blackman et al. [16], which demonstrated higher prevalence of ADHD in patients with asthma. However, the atopy of allergic disorders did not show the statistical significance between the ADHD and control group, and this result was contrary to the results of Han et al.’s study [24], which showed higher prevalence of ADHD in patients with atopic dermatitis than those without atopic dermatitis.

In this study, the lifetime prevalence rates of asthma in the ADHD group demonstrated statistical association with asthma (x^2^ = 99.09, *p =* 0.000). Also the lifetime prevalence rates of allergic rhinitis in the ADHD group demonstrated statistical association with allergic rhinitis (x^2^ = 56.08, *p =* 0.000) (Table 1). Other allergic disorders did not show statistical significance. Even though we could not clearly define a causal or order relationship between asthma and allergic rhinitis and ADHD, we demonstrate an association between them.

The exact cause of ADHD remains unknown. However, it would be valuable to identify environmental and genetic factors likely to be involved in causing ADHD [25]. In the past, it was believed that ADHD was caused by the side effects of allergic disorders: allergic reactions engender cholinergic/adrenergic activity imbalances in the central nervous system, leading to poorly regulated arousal levels of ADHD symptoms in some children [26]. In addition, the results of other studies suggested the possibility of a causal relationship between ADHD and allergic disorders, based on the fact that children with ADHD were more likely to experience symptoms of allergic disorders in similar environment [27,28]. On the contrary, other empirical studies continue to demonstrate that allergic disease and its treatment drug do not cause behavioral problems in ADHD, thus leaving the relationship between ADHD and the side effects of allergic disorders controversial [29,30].

There were few previous studies about the association asthma and ADHD. Futhermore, the explanation of the scientific evidence of the association between asthma and ADHD is very little. Even though the proof of the causal relationship between allergic disease and ADHD remains inadequate, we suspect that the existence of some pathophysiological mechanism is there on both conditions. It will be the mixture of environmental factors or genetic factors [31].

Both ADHD and allergic disease have high heritability. Genetic polymorphisms involved in dopamine neurotransmission is associated ADHD [32]. In addition to the dopamine system, the noradrenaline and histamine system are also related to ADHD [33]. “Hypersensitivity” refers to excessive and undesirable reactions produced by the normal immune system, and includes both allergic and non-allergic anaphylaxis [30]. Eighty per cent of childhood asthma may be caused by IgE-mediated and non-IgE-mediated immune reactions [30]. Even though ADHD may not be an allergic disorder, other mechanisms are assumed to be a cause of ADHD behaviors [34]. These mechanisms may be secondary to allergic immune physiology.

This study has limitations. The etiological approach to ADHD and allergic disease were not fulfilled because allergic response (immunological approach) or hypersensitivity was not evaluated. Even though it would have been meaningful to have performed a complete enumeration for the children in the twenty two elementary schools, this study was limited by the fact that all participants were surveyed from the only seven cities that may not be considered a representative of other areas. In terms of subjects, it is also problematic to generalize, since they are only first and second grade children. Other limitations include a smaller ADHD cohort compared to the control group. Although this problem may be common in a community-based study, the difference in the size of the two groups remains a limitation in this study. Another limitation being the covariance factors of allergic disorders could not be removed when the result of the study was analyzed. Besides, physical examination for allergic disorders and immunologic blood test were not performed. More concrete symptoms need to be examined to investigate the association between allergic disorders and ADHD. Finally, in order to examine ADHD as the secondary symptoms of allergic disorders, a prospective study is more appropriate than a cross-section study. Since allergic disorders and ADHD share common etiological elements, it is not clear if ADHD symptoms are the secondary symptoms of allergic disorders, such as sneezing, itching, sleep disturbance, and psychiatric and social dysfunction.

## Conclusion

We have investigated the associations between ADHD and childhood allergic disease by evaluating their respective prevalence. The lifetime prevalence rate of asthma in ADHD patients was 36.6%, compared to a prevalence of 24.3% in control subjects. The lifetime prevalence rate of allergic rhinitis in ADHD patients was 59.0%, compared to a prevalence of 47.0% in control subjects. Statistically significant difference has been found between the two groups. In the logistic regression model of the ADHD and the control group, the relative risk of asthma was 1.60 times higher, the relative risk of allergic rhinitis was 1.38 times higher which showed statistical significance. The findings of this study suggest significant association between ADHD and childhood asthma and allergic rhinitis. Therefore, appropriate evaluation and treatment are needed for asthmatic children with attention-deficit symptoms, or allergic rhinitis with ADHD. Besides, further research is needed to determine the etiological approach towards ADHD, asthma, and allergic rhinitis.

## Competing interests

The authors declare that they have no competing interests.

## Authors’ contributions

MHL and HJK were responsible for the conception, design of the study and drafted the manuscript.MHL, HJK, MH, SJY, KCP, JL, JS, CL, DK, SJH, HIC performed the data collection and MYL performed the data analysis. All authors participated in the interpretation of the findings. And all authors read and approved the final version of the paper.

## Pre-publication history

The pre-publication history for this paper can be accessed here:

http://www.biomedcentral.com/1471-244X/14/70/prepub

## References

[B1] The International Study of Asthma and Allergies in Childhood (ISAAC) Steering Committee Worldwide variation in prevalence of symptoms of asthma, allergic rhino-conjunctivitis and atopic eczema Lancet 1998 351 1225 1235 10.1016/S0140-6736(97)07302-9 9643741

[B2] Center for Disease Control and Prevention(CDC) Asthma in the U 1998 Atlanta, GA: Center for Disease Control and Prevention

[B3] American Psychiatric Association Committee on Nomenclature and Statistics Diagnostic and statistical manual of mental disorders text revision 4th ed 2002 Washington, DC: American Psychiatric Association Press

[B4] Cho SC Shin YO Prevalence of disruptive behavior disorders Korean J Child Adolesc Psychiatry 1994 5 141 149

[B5] Pyo KS Park SH Kim SH Cho YR Kim HY Moon KR The prevalence of attention deficit hyperactivity disorder in urban elementary school children 2001 Gwangju: Josun University

[B6] Eaves LJ Silberg JL Meyer JM Maes HH Simonoff E Pickles A Rutter M Neale MC Reynolds CA Erikson MT Heath AC Loeber R Truett KR Hewitt JK Genetics and developmental psychopathology: the main effects of genes and environment on behavioral problems in the Virginia twin study of adolescent behavioral development J Child Psychol Psychiatry 1997 38 965 980 10.1111/j.1469-7610.1997.tb01614.x 9413795

[B7] Biederman J Faraone S Attention-deficit/hyperactivity disorder Lancet 2005 366 237 248 10.1016/S0140-6736(05)66915-2 16023516

[B8] Fowler MG Davenport MG Garg R School functioning Of US children with asthma Pediatrics 1992 90 939 944 1437438

[B9] Lindgren S Lokshin B Stromquist A Weinberger M Nassif E McCubbin M Frasher R Does asthma or treatment with theophylline limit children’s academic performance? New Engl J Med 1992 327 926 930 10.1056/NEJM199209243271305 1513349

[B10] Bender BG Ikle DN DuHamel T Tinkelman D Neuropsychological and behavioral changes in asthmatic children treated with beclomethasone dipropionate versus theophylline Pediatrics 1998 101 355 360 10.1542/peds.101.3.355 9480997

[B11] Daly J Biederman J Bostic J Maraganore A Lelon E Jellinek M The relationship between childhood asthma and attention deficit hyperactivity disorder: a review of literature J Atten Disord 1996 1 31 40 10.1177/108705479600100103

[B12] Kashani JH Koning P Sheperd JA Wilfley D Morris DA Psychopathology and self concept in asthmatic children J Pediatr Psychol 1988 13 509 520 10.1093/jpepsy/13.4.509 3216273

[B13] Bussing R Halfon N Benjamin B Wells KB Prevalence of behavior problems in US children with asthma Arch Pediatr Adolesc Med 1995 149 565 572 10.1001/archpedi.1995.02170180095018 7735414

[B14] Jung JS Kim KH Hong KEM A study on cormorbid psychopatholgy and parenting attitude in children and adolescents with atopic dermatitis Korean J Child Adolesc Psychiatry 1999 10 34 42

[B15] Paulson JF Buermeyer C Nelson-Gray RO Social rejection and ADHD in young adult: an analogue experiment J Atten Disord 2005 8 127 135 10.1177/1087054705277203 16009661

[B16] Blackman JA Gurka MJ Developmental and behavioral comorbidities of asthma in children J Dev Behav Pediatr 2007 28 92 99 10.1097/01.DBP.0000267557.80834.e5 17435459

[B17] White BA Sander N Asthma from the perspective of the patient J Allergy Clin Immunol 1999 109 547 552 10.1016/s0091-6749(99)70273-3 10452788

[B18] Rachelefsky GS Wo J Adelson J Mickey MR Spector SL Katz RM Siegel SC Rohr AS Behavior abnormalities and poor school performance due to oral theophylline use Pediatrics 1986 78 1133 1138 3786037

[B19] Pretorius E Asthma medication may influence the psychological functioning of children Med Hypothesis 2004 63 409 413 10.1016/j.mehy.2003.12.049 15288358

[B20] Shin MS Cho SC Chun SY Hong KE A study of the development and standardization of ADHD diagnostic system Korean J Child Adolesc Psychiatry 2000 11 91 99

[B21] Oh KJ Assessment of children with attention deficit hyperactivity disorder Korean J Child Adol Psychiatr 1990 1 65 76

[B22] Kim YS So YK Noh JS Ko SG Koh YJ The reliability and validity of Korean parent and teacher ADHD rating scale J Korean Neuropsychiatr Assoc 2002 41 283 289

[B23] Lee S-I Prevalence of childhood asthma in Korea: international study of asthma and allergies in childhood Allergy Asthma Immunol Res 2010 2 61 64 10.4168/aair.2010.2.2.61 20358019 PMC2846742

[B24] Han DH Kim SH Chung US Cho JH Park JS Ahn JY Kim JW Association between atopic dermatitis and attention deficit hyperactivity disorder symptoms in Korean children Korean J Psychosom Med 2006 14 88 93

[B25] National Institute of Health Consensus Development Conference Statement Diagnosis and treatment of attention-deficit/hyperactivity disorder(ADHD) J Am Acad Child Adolesc Psychiatry 2005 39 182 193 10.1097/00004583-200002000-00018 10673829

[B26] Marshall P Attention deficit disorder and allergy: a neurochemical model of the relation between the illness Psychol Bull 1989 106 434 446 2682719 10.1037/0033-2909.106.3.434

[B27] Egger J Carter CM Graham PJ Gumley D Soothill JF Controlled trial of oligo-antigenic treatment in the hyperkinetic syndrome Lancet 1985 1 540 545 2857900 10.1016/s0140-6736(85)91206-1

[B28] Egger J Stolla A McEwen LM Controlle trial of hyposensitisation in children with food-induced hyperkinetic syndrome Lancet 1992 339 1150 1153 10.1016/0140-6736(92)90742-L 1349376

[B29] Biderman J Milberger S Faraone SV Guite J Warburton R Associations between childhood asthma and ADHD: issue of psychiatric cormobidity and familiality J Am Acad Child Adolesc Psychiatry 1994 33 842 848 10.1097/00004583-199407000-00010 8083141

[B30] Hammerness P Monuteaux MC Faraone SV Gallo L Murphy H Biderman J Reexamining the familial association between asthma and ADHD in girl J Atten Disord 2005 8 136 143 10.1177/1087054705277211 16009662

[B31] Johansson SG Bieber T Dahl R Friedmann PS Lanier BQ Lockey RF Motala C Ortega Martell JA Platts-Mills TA Ring J Thien F Van Cauwenberge P Williams HC Revised nomenclature for allergy for global use: report of the nomenclature review committee of the world allergy organization, October 2003 J Allergy Clin Immunol 2004 113 832 836 10.1016/j.jaci.2003.12.591 15131563

[B32] Faraone SV Perlis RH Doyle AE Smoller JW Goralnick JJ Holmgren MA Sklar P Molecular genetics of attention-deficit/hyperactivity disorder Biol Psychiatry 2005 57 1313 1323 10.1016/j.biopsych.2004.11.024 15950004

[B33] Schweitzer JB Holcomb HH Drug under-investigation for attention-deficit hyperactivity disorder Curr Opin Investig Drugs 2002 3 1207 1211 12211416

[B34] Warner JO Food and behavior. Allergy, intolerance or aversion Pediatr Allergy Immunol 1993 4 112 116 10.1111/j.1399-3038.1993.tb00078.x 8220798

